# Detecting and Discriminating *Shigella sonnei* Using an Aptamer-Based Fluorescent Biosensor Platform

**DOI:** 10.3390/molecules22050825

**Published:** 2017-05-17

**Authors:** Myeong-Sub Song, Simranjeet Singh Sekhon, Woo-Ri Shin, Hyung Cheol Kim, Jiho Min, Ji-Young Ahn, Yang-Hoon Kim

**Affiliations:** 1 School of Biological Sciences, Chungbuk National University, 1 Chungdae-Ro, Seowon-Gu, Cheongju 28644, Korea; smst04@nate.com (M.-S.S.); simranjeet261@gmail.com(S.S.S.); aomaya91@nate.com (W.-R.S.); 2 Technology Transfer Center, Korea Research Institute of Bioscience & Biotechnology, 125 Gwahak-Ro, Yuseong-Gu, Daejeon 34141, Korea; hcgimm@gmail.com; 3 Department of Bioprocess Engineering, Chonbuk National University, 567 Baekje-daero, Deokjin-Gu Jeonju, Jeonbuk 54896, Korea; jihomin@chonbuk.ac.kr

**Keywords:** shigellosis, aptamer-based fluorescent biosensor, whole cell-SELEX, DNA aptamer

## Abstract

In this paper, a Whole-Bacteria SELEX (WB-SELEX) strategy was adopted to isolate specific aptamers against *Shigella sonnei*. Real-time PCR amplification and post-SELEX experiment revealed that the selected aptmers possessed a high binding affinity and specificity for *S. sonnei*. Of the 21 aptamers tested, the C(t) values of the SS-3 and SS-4 aptamers (Ct = 13.89 and Ct = 12.23, respectively) had the lowest value compared to other aptamer candidates. The SS-3 and SS-4 aptamers also displayed a binding affinity (*K_D_*) of 39.32 ± 5.02 nM and 15.89 ± 1.77 nM, respectively. An aptamer-based fluorescent biosensor assay was designed to detect and discriminate *S. sonnei* cells using a sandwich complex pair of SS-3 and SS-4. The detection of *S. sonnei* by the aptamer based fluorescent biosensor platform consisted of three elements: (1) 5’amine-SS-4 modification in a 96-well type microtiter plate surface (*N*-oxysuccinimide, NOS) as capture probes; (2) the incubation with *S. sonnei* and test microbes in functionalized 96 assay wells in parallel; (3) the readout of fluorescent activity using a Cy5-labeled SS-3 aptamer as the detector. Our platform showed a significant ability to detect and discriminate *S. sonnei* from other enteric species such as *E. coli*, *Salmonella typhimurium* and other *Shigella* species (*S. flexneri*, *S. boydii*). In this study, we demonstrated the feasibility of an aptamer sensor platform to detect *S. sonnei* in a variety of foods and pave the way for its use in diagnosing shigellosis through multiple, portable designs.

## 1. Introduction

The *Shigella* species of bacteria is the causative agent of shigellosis, also known as bacillary dysentery, which has symptoms ranging from dysentery, tenesmus, and abdominal cramps to diarrhea with blood and mucous [1]. It is estimated that more than 165 million cases are reported annually and approximately one million people die from shigellosis worldwide each year [2]. The *Shigella* genus is comprised of four species: *Shigella dysenteriae*, *Shigella flexneri*, *Shigella boydii*, and *Shigella sonnei*. *S. flexneri* is the most common species in developing countries whereas *S. sonnei* is the predominant species in industrialized countries [3,4]. Several reports have shown that most shigellosis outbreaks are closely related to an influx of people with a history of international travel, which is difficult to control due to their low infectious dose [5,6]. Moreover, since shigellosis tends to spread very rapidly by mass feeding in closed and concentrated groups, such as army barracks and schools [7], there is an urgent need for rapid, simple, and specific detection methods for shigellosis.

The conventional plate-based method for detecting foodborne pathogens has the limitations of being time-consuming and requiring selective media. *Shigella* species are fastidious bacteria that survive poorly outside the human body, therefore plate-based methods can detect only a small fraction of shigellosis cases [8]. To overcome the limitations of the conventional culture method, rapid molecular diagnostic methods like real-time PCR [9] and PCR-ELISA [10] have been introduced. Although molecular detection methods using virulence genes are highly sensitive, they still have several limitations: (1) non-specific amplification and primer dimerization, (2) failure to recognize bacteria serotypes and antibiotic resistance (3) requirement of specialized lab equipment such as gel-electrophoresis apparatuses and/or antibodies.

More recently, many biosensor approaches for detecting pathogenic bacteria have been investigated, and this biosensor platform offers the advantage of label-free, real-time, and visible detection [11,12]. As an ideal element for recognition, specific aptamers in bacteria have been increasingly targeted over the use of antibodies in a variety of analytic technologies. Several efforts have been reported, such as the Surface Plasmon Resonance (SPR) analysis [13], aptamer-based lateral flow dipstick tests [14], cross-linked hydrogels [15], aptablotting assay [16].

An aptamer is an artificial single-stranded oligonucleotide that can be specifically isolated through the Systematic Evolution of Ligands by exponential enrichment (SELEX) process and capable of recognizing a variety of targets in systemically designed detection platforms [17,18,19,20]. Some studies have reported ingenious efforts to combine the recognition ability of aptamers with advanced nanomaterials in the field of drug delivery and targeted therapeutics because of their strong stability under a wide range of temperature, pH and in-vivo/ex-vivo conditions [21,22]. In a recent study, a sandwich-based detection method using dual aptamers to recognize *S. sonnei* has also been reported [23]. Herein, we present a specific aptamer sandwich pair against *S. sonnei* in a fluorescence detection platform. We have carried out a Whole-Bacteria SELEX process to obtain single stranded DNA aptamers with high affinity and specificity for *S. sonnei*. Aptamers were selected using whole live *S. sonnei* cells as targets, while *E. coli* cells were used as a negative control. After 10 rounds of selection, the aptamer pair, SS-3 and SS-4 were chosen and designed as the detection and capture probe in our aptamer-based fluorescent biosensor platform. Our platform allow for the rapid and sensitive discrimination between *S. sonnei* and other enteric bacteria, such as *E. coli*, *Salmonella typhimurium* and other *Shigella* species (*S. flexneri*, *S. boydii*).

## 2. Results

### 2.1. Whole Bacteria Cell SELEX for the Isolation of S. sonnei Specific DNA Aptamers 

**Figure 1:**
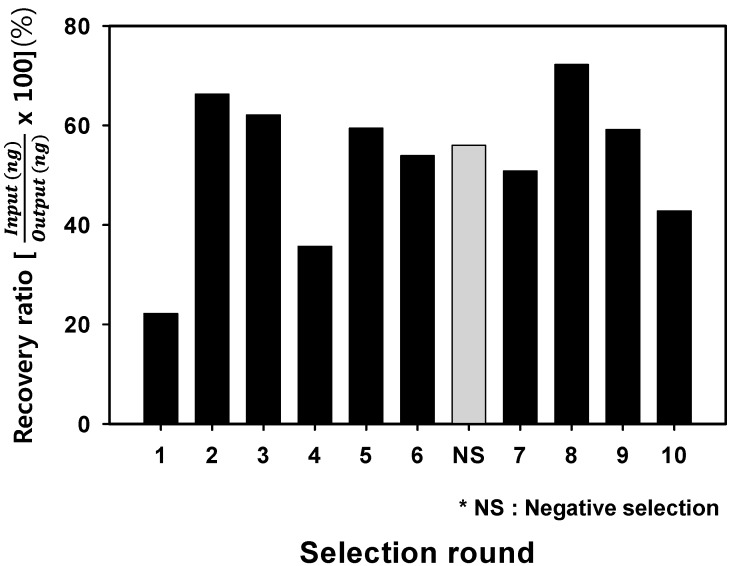
Measurement of eluted aptamers during the SELEX processes. The ssDNA was obtained from each selection round. The recovery ratio was calculated using the amount of input and output DNA concentration through the 10th round of the SELEX processes. The negative selection round (NS) was performed after round 6 to prevent enrichment of non-specific binding aptamers.

The randomized oligonucleotide aptamer library, containing about 10^16^ diverse sequences, was initially introduced by incubating cultured live *S. sonnei* cells. In each round of selection against *S. sonnei*, the concentration of bound DNA species was increased to further drive selection toward a high-affinity and high-specificity aptamer pool. The initial oligonucleotide library consisted of a 40 nucelotide randomized region (N_40_), and primer binding sites were chemically synthesized. After 10 rounds of SELEX, nanodrop spectrophotometer analysis was performed to monitor and assess the binding affinity of aptamer sub-pools to *S. sonnei*. To eliminate non-specific and weakly binding DNA aptamers, we incorporated a negative-selection step using *E. coli* cells after the 6th selection round. It is important to note that gram-negative *E. coli* is closely related to *Shigella* spp. and discriminating *Shigella* from *E. coli* is clinically necessary, since *Shigella* is a source of infectious diarrhea which can cause a worldwide outbreak [24,25]. Genetically, the chromosomal structure of *S. sonnei* has the same replication origin and terminus as *E. coli* [26,27]; this means not only they evolutionarily related, but they most likely utilize the same cellular machinery in both DNA replication and in the gene expression/regulation processes for extracellular structure. As described in Figure 1, during the SELEX process, non-specific and/or weakly binding aptamer candidates were efficiently excluded using the negative count-partner, *E. coli*, in the 6th round. Thus the selected cell-specific aptamers can specifically recognize the outer-cellular targets of *S. sonnei* and can be used as high affinity binding probes in the detection platform. To determine if the DNA aptamer against *S. sonnei* cells would be enriched during the SELEX process, the amount of recovered ssDNA from each selection round was analyzed. The eluted concentration was highest in the 8th round after the negative selection (6th) round and 7th selection round. These results indicate that the ssDNA aptamer candidates were successfully enriched for whole live bacterial cells during the process. A decrease in ssDNA concentration was also observed after the negative selection round due to the removal of unbound ssDNA. We therefore determined that the 8th selection round is the optimal state to isolate appropriate aptamer candidates.

### 2.2. Analysis of Optimal Enrichment States for Isolating Aptamers 

The retrieval concentration pattern showed that the overall tendency to increase with each selection round progressed until the 8th round, after which it decreased. This implies that the cell specific aptamers were saturated and enriched during the SELEX processes. To further demonstrate the difference in binding ability round by round, we employed real-time PCR (RT-PCR), which proved valuable in validating the optimal state of DNA aptamer enrichment [20].

Potential aptamer pools collected from the initial (0 R), 7th, 8th, and 9th round were incubated with *S. sonnei* cells (1.2 × 10^8^ cfu/mL) in parallel for 1 h. After washing three times, bound DNA was recollected. The recovered DNA samples were each amplified using an optimized RT-PCR cycle and the fluorescent signal was monitored by the MiniOpticon^TM^ Real-time (RT)-PCR fluorescent signal detection system (Bio-Rad, CA, USA). The PCR experiment was independently conducted in triplicate and data were analyzed to obtain the average C(t) values (Figure S1). The samples from the 8th round SELEX showed a lower C(t) (*p* < 0.001), C(t) = 13.66, as compared to other samples. The C(t) for the 7th and 9th rounds were 14.33 and 13.85, respectively. Based on these RT-PCR experiments, it was clear that the sample collected from the 8th round pool did show a higher amount of DNA aptamers for *S. sonnei* cells.

To isolate potential aptamers that recognize whole live *S. sonnei* cells, the DNA aptamer pool from the 8th selection round was cloned, followed by the sequencing of 40 clones. By multiple sequence alignment using the ClustalX software program, a total of 21 aptamers (SS-1 to SS-21) with intact random sequences were obtained (Table S1).

### 2.3. Binding Test of Aptamer Candidates by Post-SELEX

**Figure 2:**
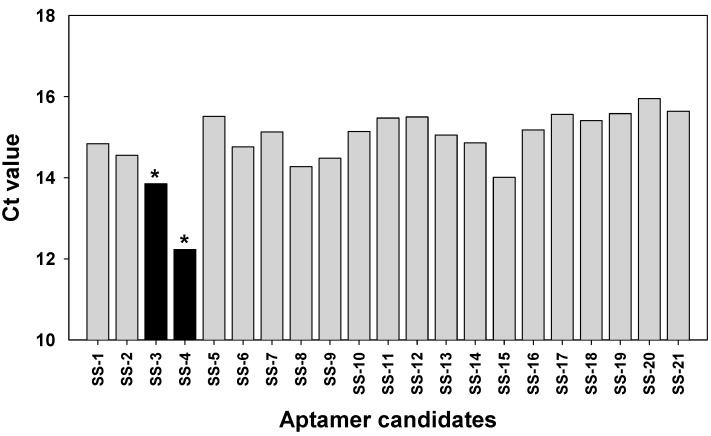
Characterization of binding aptamers. Twenty-one aptamer candidates (SS-1 to SS-21) were analyzed by a post-SELEX method as described in Materials and Method. After incubation with *S. sonnei* cells, aptamers were retrieved. The eluents were amplified by RT-PCR and the average C(t) values were analyzed in triplicate individual experiments. *: C(t) value < 14.

**Figure 3:**
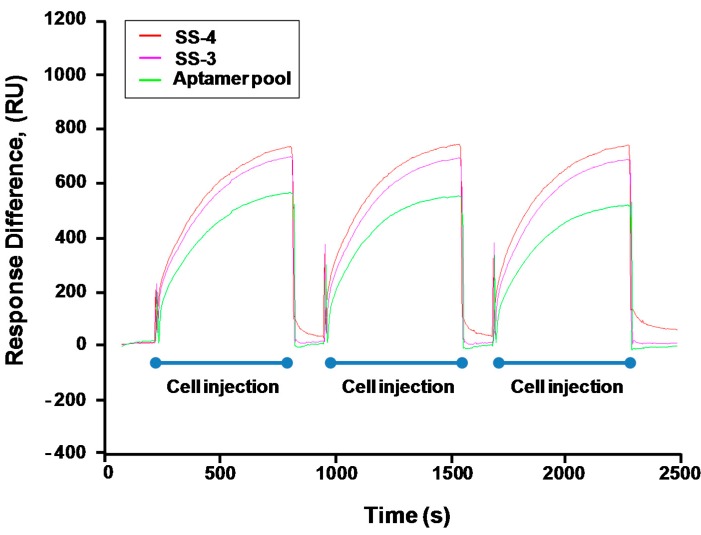
Repeatability of aptamer: Aptamer reusability was tested by removing target cells from the aptamer immobilized layer using NaCl treatment and injecting right after the target cells. The aptamer binding property was not altered during the consecutive interaction cycles with *S. sonnei* (4 × 10^8^ cfu/mL) and regeneration in flow.

The binding value for each apamers was determined by analyzing the SPR and C(t) value using RT-PCR after the post-SELEX experiment (Figure S1 and Table 1). For the C(t) value analysis, 180 pmol of each aptamer was incubated with *S. sonnei* cells (4 × 10^8^ cfu/mL) at 4 °C for 1 h in 200 μL. After washing, each aptamer-cells complex was prepared for PCR amplification. The precipitated aptamers with cells were recovered and mixed with primers and PCR mixtures. Expecting that all of the unhybridized or weakened DNA aptamers would be eliminated by washing three times with 500 μL of TE buffer in this post-SELEX step, the C(t) value by RT-PCR analysis showed which aptamer could tightly bind to their target cells. Of the 21 aptamers tested, results in Figure 2 show that the C(t) value of SS-3 and SS-4 aptamers was significantly lower (Ct = 13.89 and Ct = 12.23, respectively) than that obtained when the other aptamers were used (Ct > 14). It is understandable that most of the 19 aptamers were weakly bound to *S. sonnei* cells, whereas SS-3 and SS-4 aptamers showed tight binding to their target cells. Binding ability (SS-3 and SS-4) for detecting whole live *S. sonnei* was then evaluated by performing SPR assays with a nonspecific control (random aptamer pool) at the same cell concentration. The biotinylated ssDNA aptamers (SS-3, SS-4 and the random aptamer pool) were coupled to the streptavidin sensor chip. The whole cell binding experiment was carried out in an activation buffer (HBS-EP) solution. It was confirmed that significant aptamer binding signal came from the recognition of *S. sonnei*. No significant binding was observed in the random aptamer pool. Aptamer repeatability was also tested by removing target cells from the aptamer immobilized layer using NaCl treatment and then reinjecting the target cells. The complete detection cycle was observed without altering the aptamer’s binding property during the consecutive interactions with *S. sonnei* (4 × 10^8^ cfu/mL) and regeneration in flow (Figure 3). The SS-3 and SS-4 aptamers displayed binding affinity (*K_D_*) of 39.32 ± 5.02 nM and 15.89 ± 1.77 nM, respectively (Table 1). In order to compare the secondary structures of the two aptamers, SS-3 and SS-4, we used the web-based tool Mfold (http://mfold.bioinfo.rpi.edu/cgi-bin/dna-form1.cgi). The results of both the sequences and secondary structures of the SS-3 and SS-4 aptamers are given in detail in Table 1. No consensus was found between SS-3 and SS-4.

### 2.4. Detection and Discrimination of S. sonnei by Aptamer-Based Fluorescent Biosensor

**Figure 4:**
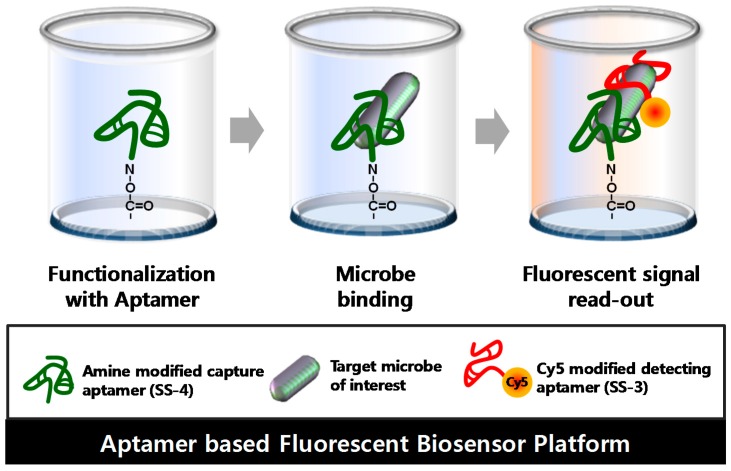
Principle of *S. sonnei* detection using an aptamer-based fluorescent biosensor platform. An aptamer-based fluorescent biosensor platform comprised of three major factors: the functionalized surface by the capture aptamer (SS-4), target binding, and signal read-out by the Cy5-labelled detection aptamer (SS-3).

An aptamer-based fluorescent biosensor assay has been designed to detect and discriminate *S. sonnei* cells using a sandwich complex pair of SS-3 and SS-4. The principle of the biosensor strategy is illustrated in Figure 4. First, a carboxyl group modified 96-well type microtiter plate surface (NOS chip surface) was activated using 5’amine-SS-4 as the capture probes. *S. sonnei* and test microbes were then added to the functionalized well in parallel. Finally, the capture signal with fluorescence activity was reported by SS-3, the Cy5-labeled detection aptamer. In detail, the surfaces were first functionalized by using an aptamer solution with a concentration of 1 μM (100 pmol per 100 μL of treatment volume) per well. If fact, although 1 μM of the 5’ amine modified aptamer (SS-4) was used to covalently bound to the NOS-well surface, it was necessary to determine the actual amount of apatmer bound to each well of the microplate.

**Figure 5:**
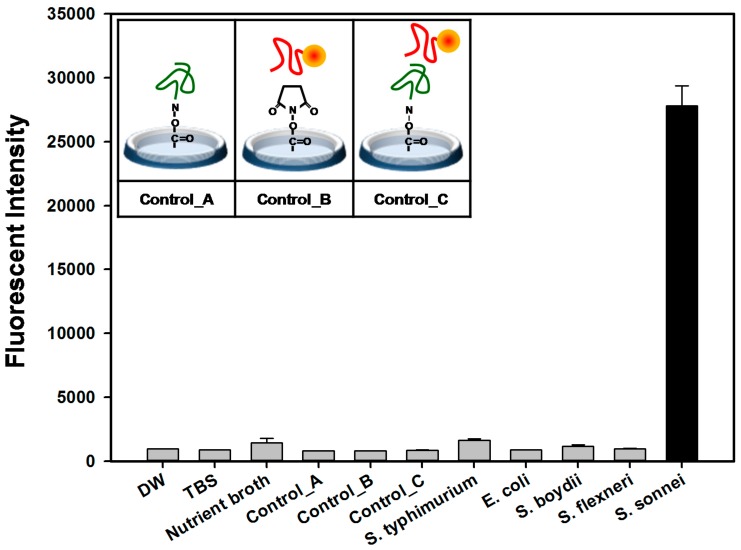
Sandwich binding activity of *S. sonnei*. Fluorescence intensity with three controls (A: basal signals of the plate surface with the detection aptamer; B: signals without the capture aptamer; C: signals without the target cells in sandwich complex) was determined by using an aptamer based fluorescent biosensor assay.

Sensor wells were then challenged with different bacterial cells (*S. flexneri* (KCTC 2517), *S. boydii* (KCTC 22528), *E. coli* (KCTC 1116), and *S. typhimurium* (KCTC 2053)). The strains were diluted in commercially available bottled water (4 × 10^8^ cfu per mL). After incubating with 500 pmol of the Cy5-labeled SS-3, the fluorescence intensity detected from the microtiter plate was recorded at λex = 646 nm and λem = 662 nm. We observed significantly increased fluorescence intensity value only in the *S. sonnei* cells as shown in Figure 5. Supplementary Figure S2 shows the intensity of the fluorescent signal, which represents the aptamer SS-4/target cells/SS-3-cy5 complex. A linear dynamic relationship between the aptamer sensor and the cells (within the range of 10^3^–10^6^ cells per mL) is seen, and at over 10^7^ cells binding signals were saturated.

As control experiments, the activated and functionalized surface by capture aptamer (control_A), the carboxyl group coated microtiter plate surface (control_B), and aptamer-aptamer binding without bacteria cells (control_C) were compared simultaneously. *S. sonnei* detection by aptamer sandwich pairs in our aptamer-based fluorescent biosensor platform was clearly distinguishable from the controls (A to C), nutrient broth (LB), buffer solution (TBS), and DW.

**Figure 6:**
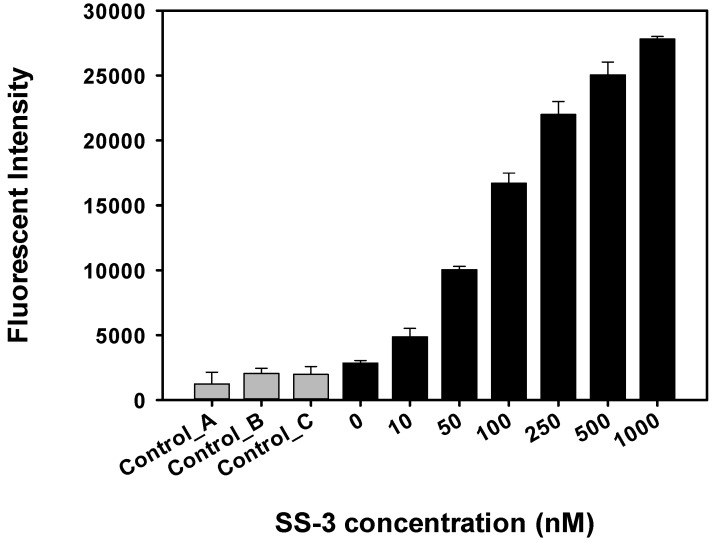
Sandwich binding activity of *S. sonnei*. Various concentrations of SS-3 were used. Fluorescence intensity with three controls (A: basal signals of the plate surface with the detection aptamer, B: signals without the capture aptamer, C: signals without the target cells in sandwich complex) was determined by using an aptamer- based fluorescent biosensor assay.

Concentration dependent aptamer binding activity against *S. sonnei* was also tested on the sandwich sensor assay. Results in Figure 6 show that SS-3 binds to target cells in a concentration-dependent manner.

These results indicated that competitive binding between SS-3 and SS-4 was not observed. Therefore, SS-3 and SS-4 are assumed to have different binding sites on the surface of *S. sonnei.* Supplementary Figure S2 shows the reliable and sensitive quantification of *S. sonnei* with a linear dynamic range of 10^3^–10^6^ cells/mL (R^2^ = 0.97), and a limit of detection of 10^3^ cells per mL.

## 3. Discussion

Many types of *S. sonnei* detection methods have been developed over the last several decades, including immunosorbent assays (ELISA) and polymerase chain reaction (PCR) amplification analysis. However, ELISA and PCR-based amplification assays have a high risk of false-positives and false-negatives due to the inhibition by the cellular component itself and the clinical sample matrix. Moreover, ELISA requires labeled primary and/or secondary antibodies. There is considerable cross-reactivity in the use of antibodies which can recognize Gram-negative bacteria serotype O antigens since many O groups of *E. coli* are cross-reactive or identical with specific O regions of *Shigella* [28]. In this paper, an aptamer-based fluorescent biosensor platform was developed and evaluated for critical discrimination of *S. sonnei* cells from other enteric pathogens. The biosensor platform consisted of a molecular recognition element pair, amine-modified capture aptamer (SS-4) and fluorescent labeled Cy5-detecting aptamer (SS-3). These aptamers were efficiently selected by Whole Bacteria-SELEX (WB-SELEX). A key aspect of the SELEX experiment was the recognition of the optimal enriched round during the process.

In fact, SELEX process can be modified in a variety ways to improve the aptamer specificity including the participation of counter-/negative selection and the incorporation of stringent buffer conditions during each round [29]. Here we used a post-SELEX procedure and real-time PCR analysis to obtain a highly efficient selection of aptamers. The counter-partner bacteria *E. coli*, which is closely related to *S. sonnei* in evolution, was used for negative selection of non-specific aptamers. A significantly increased intensity of fluorescence was observed for only the *S. sonnei* cells, not *S. flexneri* or *S. boydii*.

Our aptamer sensor platform therefore exhibits a high specificity that may be due to the formation of secondary and tertiary structures against a certain extracellular component of *S. sonnei*. In particular, the aptamer provides a clear modification mechanism for integrating it onto a solid surface within the sensor platform. This allows a specific and unique orientation to detect targets when anchored to the surface. This approach has the potential to be utilized in a direct sample-to-result detection of pathogens in multiple, portable designs. It may also represent an important development in diagnostics and therapeutics involving other pathogens by simply substituting aptamers.

## 4. Materials and Methods 

### 4.1. Bacterial Strains and Cell Maintenance 

*Shigella sonnei* (KCTC 2518), *Shigella flexneri* (KCTC 2517), *Shigella boydii* (KCTC 22528), *Escherichia coli* (KCTC 1116), and *Salmonella typhimurium* (KCTC 2053) were obtained from the Korean Collection for Type Cultures (KCTC, Jeongeup-si, South Korea). The media for bacterial culture were: nutrient broth (BD Difco, Oxford, UK) for *S. sonnei*, *S. flexneri*, and *S. typhimurium*; Tryptic Soy Broth (BD Difco) for *S. boydii*; Luria-Bertani media (LB; BD Difco) for *Escherichia coli*. All bacteria were cultured at 37 °C with shaking at 180 rpm.

### 4.2. Whole SELEX Pprocedure

The initial oligonucleotide library consists of a 40-nucleotide randomized region (N_40_) and primer binding sites was chemically synthesized (Bioneer, Daejeon, Korea): 5′-ATA CCA GCT TAT TCA ATT -N_40_- AGA TAG TAA GTG CAA TCT-3′. The random library was amplified by PCR using forward primer (5′-ATA CCA GCT TAT TCA ATT) and biotinylated reverse primer (5′-AGA TTG CAC TTA CTA TCT-3′) during the selection process. PCR product was purified using the QIAquick PCR purification kit (Qiagen, Germantown, MD, USA) and single-stranded DNA (ssDNA) was generated by removing the bionitylated double-stranded DNA (dsDNA) using streptavidin agarose resin (Pierce, WA, USA). The resulting ssDNA was confirmed by comparison with double-stranded DNA (PCR product) on 10% polyacrylamide gel containing 0.5X TBE buffer (Thermo Fisher Scientific Korea, Seoul, Korea). The ssDNA library (800 pmol) was then heat denatured for 5 min at 85 °C and renatured for 2 h at room temperature (RT) to allow the formation of a stable structure.

The subculture of *S. sonnei* (4 × 10^8^ cfu/mL) was washed with TBS buffer (10 mM Tris-HCl, 0.85% NaCl, pH 8.0) and cell pellets were incubated with structured ssDNA library (800 pmol) at 4 °C with gentle shaking for 1 h in 200 μL nutrient broth. Following incubation, the mixture was centrifuged (3500 rpm at 4 °C for 5 min) and washed with TBS buffer to remove any unbound or weakly bound aptamers. To elute cell-bound aptamers, the cells were re-suspended in the TE buffer (10 mM Tris-HCl, 1 mM EDTA, pH 8.0), heated at 85 °C for 5 min, snap-cooled on ice, and centrifuged. The eluted ssDNA in supernatant was recovered by phenol/chloroform extraction and ethanol precipitation and then used as the template DNA for the next round of selection. The resulting purified aptamers were run on a 1% agarose gel to confirm correct DNA size. To increase the specificity of aptamers candidates, negative selection was performed after the 6th round. The 6th sub-library of ssDNA was incubated with *E. coli* as a counter-partner at 4 °C for 1 h in LB broth, and unbound aptamers in the supernatant were collected for the next round of selection.

### 4.3. Real-Time PCR Analysis 

To determine the optimal selection round, the eluted ssDNA concentrations from each selection round were analyzed. The ssDNA concentrations from each round were quantified by measuring the absorbance at 260 nm using a nanodrop spectrophotometer (Thermo Fisher Scientific Korea Ltd., Seoul, Korea). The binding DNA aptamer pool from the initial round, 7th, 8th, and 9th round were prepared using the same concentration (360 pmole) and incubated with target cells (1.2 × 10^8^ cfu/mL) in parallel for 1 h. After three times washes, bound ssDNA was retrieved from the cell-pool complexes. Serial dilutions of each samples (1× –no dilution, 10×, 100×) were prepared and analyzed through real-time PCR using the MiniOpticon^TM^ Real-time PCR fluorescence signal detection system (Bio-Rad, Hercules, CA, USA). For the amplification, 20 μL of reaction mixtures were prepared consisting of 10 μL of 1X iQ SYBR Green Supermix (Bio-Rad), 8.8 μL of PCR water, 0.1 μL of 0.1 mM of forward primer, 0.1 μL of 0.1 mM of reverse primer, and ten-fold serial dilution of each template DNA (1 μL). The PCR parameters were: taq activation (3 min at 94 °C); 40 cycles of PCR at 94 °C for 30 s, at 52 °C for 30 s, and at 72 °C for 15 s; followed by 5 min of extension at 72 °C. All sample analyses were performed in triplicate. Efficiency measurement and threshold cycle (Ct) analysis were performed using the MJ opticon monitor analysis software, version 3.1 (Bio-Rad). The DNA pool from the optimal round (8th round) was cloned into the T-blunt vector in *E. coli* DH5α (Solgent, Daejeon, Korea). The purified plasmids from individual white colony were analyzed by sequencing and ClustalX sequence alignment program [30].

### 4.4. Post-SELEX for the Binding Affinity Test

To further confirm the binding affinities of the aptamer candidates to *S. sonnei*, a post-SELEX step was conducted. A total of 21 aptamer candidates with the same concentration (180 pmole) were prepared and incubated with target cells (1 × 10^8^ cfu/mL) in parallel. After the post-SELEX process, each aptamer candidates was analyzed through real-time PCR as described above. The quantity of amplicons were analyzed using MiniOpticon^TM^ Real-time PCR fluorescence signal detection system (Bio-Rad) by comparing the C(t) values. The secondary structures of the final selected *S. sonnei* binding aptamers were predicted by free energy minimization algorithm using *Mfold*. (http://mfold.bioinfo.rpi.edu/cgi-bin/dna-form1.cgi).

### 4.5. Binding Activity of Selected Aptamers 

We used the streptavidin immobilized sensor chip SA (for the Biacore 3000, GE, Buckinghamshire, UK) for the interaction test. The 5′-biotin-modified DNA aptamers for *S. sonnei* (SS-3 and SS-4) were immobilized by passing 500 nM aptamers for 10 min at 10 µL per min on SA chip. The SA chip was pre-activated with 1 mL of HBS-EP buffer (GE Healthcare, Buckinghamshire, UK) at a flow rate of 10 µL per min for 7 min then 1 mL of the running buffer at a flow rate of 10 µL per min for 10 min. Next, the 5′-biotinylated aptamers were immobilized on the chip as recommended in the manufacturer’s manual. To monitor the consecutive interaction, we used the Biacore instrument, and all procedures were automatically implemented to create repetitive cycles of sample injection (90 µL injection samples, at a flow rate of 10 µL per min) and regeneration (1 M NaCl, 50 mM NaOH, 50% isopropanol), according to the instruction guidelines (BIA evaluation program, version 4.1, GE Healthcare, Buckinghamshire, UK).

### 4.6. Sensitivity Detecting of S. sonnei by Aptamer-Based Fluorescent Biosensor Assay

Aptamer-based fluorescent biosensor assays were performed using a 96 well microplate with covalently-linked N-oxysuccinimide (NOS) esters that quickly react with primary amine groups (Corning Inc., Corning, NY, USA). In order to varify the aptamer’s ability to distinguish *S. sonnei* from other food-poisoning bacteria (other *Shigella* species, *E. coli*, *S. typimurium*), capture probes and detection probes were chemically synthesized as 5′-amine-modified SS-4 and Cy5-labelled SS-3 at the 5′-end respectively. The assay was prepared as follows: (1) the 5′ amine modified aptamer (SS-4) was coated with a concentration of 1 μM (100 pmol in 100 μL treatment volume) per single well NOS (N-oxysuccinimide) surface in a 96-microwell plate for 1 h at RT. After immobilization, the NOS surfaces were washed 3 times with 0.1% Tween-20 in TBS (TBST), after which 100 μL of blocking buffer (2% BSA in sodium phosphate buffer); (2) To prepare the assay sample, five different bacterial species were spiked into commercially available bottled water with an optical density at 600 nm (OD_600_=0.8, 4 × 10^8^ cfu/mL). Next, 100 μL of each bacteria samples was added into the wells and incubated with gentle shaking for 1 h at RT. The unbound cells were removed, and the plates were washed twice with TBST; (3) Finally, 1 μM of the Cy5-labeled detecting aptamer (SS-3) was introduced into the individual wells and incubated at RT for 30 min. Following incubation, unbound Cy5 aptamers were washed with TBST, and the absorbance was measured at 650 nm using the SpectraMax M2 multi-detection microplate reader (Molecular Devices, Sunnyvale, CA, USA) at excitation/emission wavelengths of 646/662 nm respectively.

## 5. Conclusions

In summary, aptamer-based fluorescent biosensor platform to detect *S. sonnei* was developed using a Whole-Bacteria SELEX (WB-SELEX) strategy. We used a post-SELEX procedure and real-time PCR analysis for the highly efficient selection of aptamers. Our platform shows a significant ability to detect and discriminate *S. sonnei* from other enteric species such as *E. coli*, *Salmonella typhimurium* and other *Shigella* species (*S. flexneri*, *S. boydii*). The sensitive quantification of *S. sonnei* with a linear dynamic range of 10^3^–10^7^ cells per mL was also possible using this platform.

## Figures and Tables

**Table 1:** Sequence of the isolated aptamer candidates.

Clone	Selected Sequence	Size (bp)	Secondary Structure	Chemical Modification	*K_D_* (nM)
SS-3	ATACCAGCTTATTCAATT CCATGGTCCCTCGTGTTTAT TATGTTGTCTGAACTGGCTG AGATTGCACTTACTATCT	76	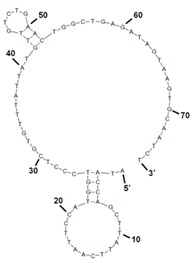	Cy5 for detection probe	39.32 ± 5.02
SS-4	ATACCAGCTTATTCAATT CCACACATACCAAAAACACA GCACACTTCATCAATTTCACG AGATTGCACTTACTATCT	77	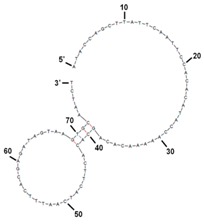	Amine for capture probe	15.89 ± 1.77

## Supplementary Materials

Supplementary materials are available online.
