# Supplementary material for: Detecting and Discriminating Shigella sonnei Using an Aptamer-Based Fluorescent Biosensor Platform
Source: Molecules. 2017 May 17;22(5):825. doi: 10.3390/molecules22050825 (PMC6154610; doi:10.3390/molecules22050825)
Supplement: Supplementary file 1 [file molecules-22-00825-s001.pdf]

[Supporting Information]

Detecting and Discriminating Shigella sonnei using Aptamer-based Fluorescent Biosensor Platform

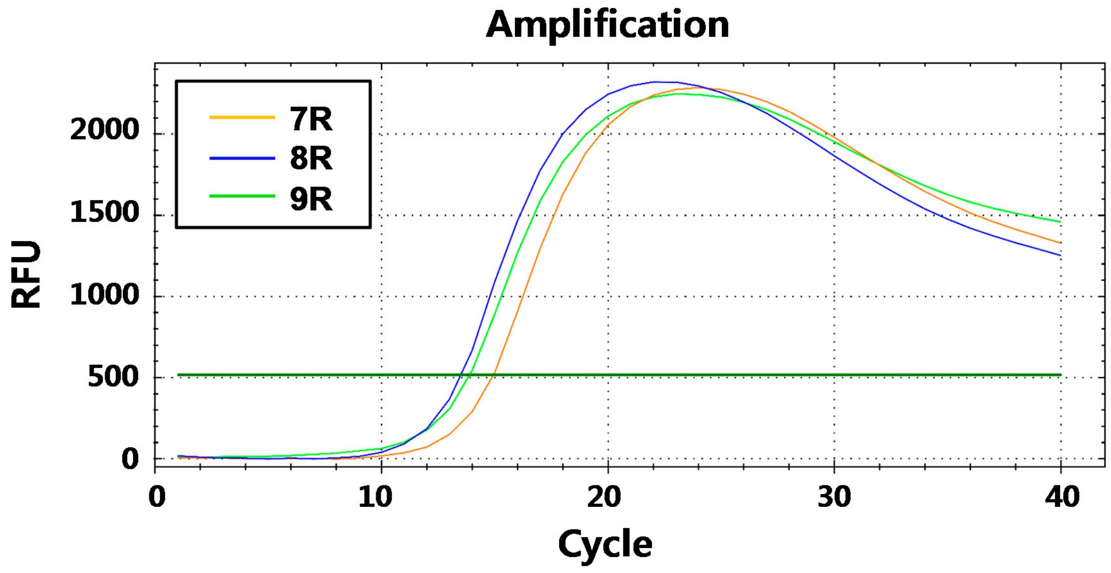

	7th Round	8th Round	9th Round
PCR Efficiency (%)	76.97	79.35	73.44
Ct value	14.33	13.66	13.85

Figure S1. Monitoring of the optimum enrichment status for isolating aptamers. PCR efficiency and C(t) values were measured using the MJ opticon monitor analysis software, version 3.1 (Bio-Rad, USA).

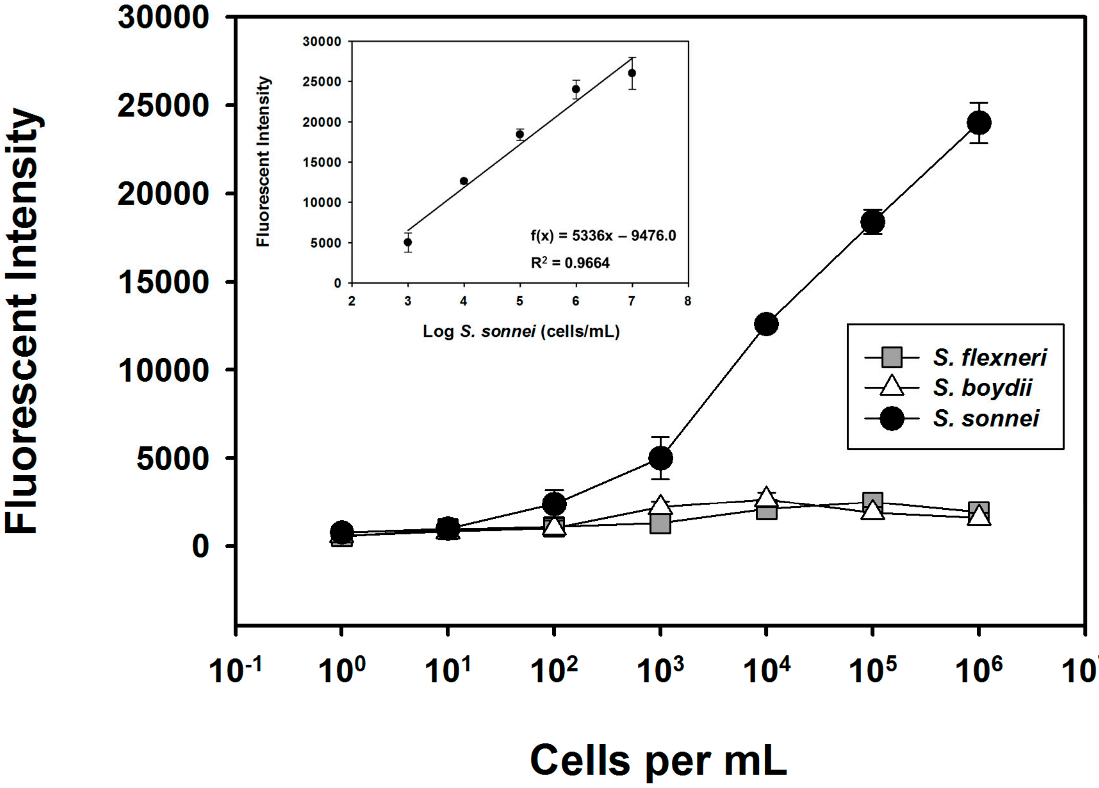

Figure S2. Sandwich binding was tested with different concentrations of *S. sonnei* ($10^0 \sim 10^7$ cells). Sandwich aptamer sensors bind spedifically to *S. sonnei*.

Table S1. Sequence of the isolated aptamer candidates

Clone	Selected sequence	Size(bp)
SS-1	GGGCATGTGGACCTGCGATTTCGGTTTGGTGTGGTTGGGG	40
SS-2	CCCCATGTCTGTTTCTTTTAACAGGTAATCCGCCTTATGC	40
SS-3	CCATGGTCCCTCGTGTTTATTATGTTGTCTGAACTGGCTG	40
SS-4	CCACACATACCAAAAACACAGCACACTTCATCAATTTCACG	41
SS-5	CAGGACAAAAATTCGGGAAGGCGGCCTTCCACTCTTTCTG	40
SS-6	ATACAGTGAAGGCCCAGGAGGCAAATTCTAGGACGAAAGC	40
SS-7	CACCAAGTCGCCTCCTCTGCCCCCATGTAAATCGTTACAT	40
SS-8	GAACAATTTCACGCACACCGCGTAGATACCACCTCGTGCA	40
SS-9	CGACGAACTACACCACAGGCCCTTAACACATCTACTTGTA	40
SS-10	CATGCTAACTGCCATCACCACTATTTATGATTCCATCCAT	40
SS-11	CGCAACACAGAAACACCCGTACACAAACAGTTTCAGCCCG	40
SS-12	TGCGTAGTTCTCTTAACAACCATTCAGCGTATTTTCGTGG	40
SS-13	CAGGCACGAGTTCATCACACACCATACACAGTTCGTTACT	40
SS-14	CCACACCACACACTACACGCATAAAACCACGAAACTACAC	40
SS-15	GGGTATGTGGACTTGCGATTTCGGTTTGGTGTGGTTGGGG	40
SS-16	CATACAGTGGCCAGATTTACCAACACACCTTTCCATACGC	40
SS-17	CCACTCACATATTAACAACACCACATGAATATATTTCG	38
SS-18	ACAGGGCATGTCTTCATCAACGCTTACCACACACCGCTCG	40
SS-19	CACTCATGCGCACCGCGTCGCACTTAATCAGTGTTGTGC	39
SS-20	GGGCATGTGGACTTGCGATTTCGGTTTGGTGTGGTTGGGG	40
SS-21	CAGACACACCAACGCACGGGCACACGCTACAAATGTAAGT	40
